# Effects of Gate Stack Structural and Process Defectivity on High-*k* Dielectric Dependence of NBTI Reliability in 32 nm Technology Node PMOSFETs

**DOI:** 10.1155/2014/490829

**Published:** 2014-08-17

**Authors:** H. Hussin, N. Soin, M. F. Bukhori, S. Wan Muhamad Hatta, Y. Abdul Wahab

**Affiliations:** ^1^Department of Electrical Engineering, University of Malaya, 50603 Kuala Lumpur, Malaysia; ^2^Faculty of Electrical Engineering, Universiti Teknologi MARA, 40450 Shah Alam, Malaysia; ^3^Department of Electrical, Electronics & Systems Engineering, Faculty of Engineering and Built Environment, Universiti Kebangsaan Malaysia, 43000 Bangi, Malaysia

## Abstract

We present a simulation study on negative bias temperature instability (NBTI) induced hole trapping in *E*′ center defects, which leads to depassivation of interface trap precursor in different geometrical structures of high-*k* PMOSFET gate stacks using the two-stage NBTI model. The resulting degradation is characterized based on the time evolution of the interface and hole trap densities, as well as the resulting threshold voltage shift. By varying the physical thicknesses of the interface silicon dioxide (SiO_2_) and hafnium oxide (HfO_2_) layers, we investigate how the variation in thickness affects hole trapping/detrapping at different stress temperatures. The results suggest that the degradations are highly dependent on the physical gate stack parameters for a given stress voltage and temperature. The degradation is more pronounced by 5% when the thicknesses of HfO_2_ are increased but is reduced by 11% when the SiO_2_ interface layer thickness is increased during lower stress voltage. However, at higher stress voltage, greater degradation is observed for a thicker SiO_2_ interface layer. In addition, the existence of different stress temperatures at which the degradation behavior differs implies that the hole trapping/detrapping event is thermally activated.

## 1. Introduction

Significant progress has been made in integrating hafnium dioxide- (HfO_2_-) based dielectrics into advanced deep-submicron metal-oxide-semiconductor field effect transistor (MOSFET) devices, replacing conventional SiO_2_ dielectrics. This development has been primarily motivated by the advantage of high-*k* materials to yield larger gate capacitance with less leakage current, which results in enhanced drivability and transistor performance in complementary metal-oxide-semiconductor (CMOS) technology. However, high-*k*-specific issues remain with regard to mobility degradation, compatibility with metal gate, and threshold voltage instabilities associated with defects, traps, and charges inside the high-*k* layers and SiO_2_ interfacial layer [1–3]. One of the most critical reliability issues is the negative bias temperature instability (NBTI), which is exacerbated by the introduction of high-*k* gate dielectrics. NBTI degradation mechanism is broadly known to involve hole captures in the high-*k* oxide *E*′ centers and generation of interface trap, *N*_it_, under a negative bias gate voltage stress, which leads to threshold voltage shift, Δ*V*th [4, 5]. This stress-induced Δ*V*th may include contributions from both the trap centers of HfO_2_ and within the interfacial layer (IL) [6]. Electron spin resonance measurements indicate the existence of trapping centers in the bulk HfO_2_ and centers within the Si/dielectric IL [7, 8]. Therefore, understanding the exact mechanism of the hole trap transformation process in each layer of the gate stack is important. How the variation in thickness of the HfO_2_ metal gate stack PMOSFETs affects the kinetics of *E*′ centers and generation of interface traps has not been thoroughly investigated. The resulting findings could help device designers minimize BTI-related degradations and gate leakages in subsequent products.

The density of *E*′ centers in the layer largely depends on the processing temperature of SiO_2_ [8, 9]. The oxygen transport between high-*k* and SiO_2_ interface has been investigated in a study on the effects on *V*_fb_ roll-off in scaled SiO_2_ IL [9]. Oxygen-deficient silicon sites, *V*_O_^+^, are generated as the SiO_2_ IL thickness is increased, which then reduces the threshold voltage of the transistor. Considering the high density of *E*′ defects in the SiO_2_ IL, the IL has been suggested as an oxygen-deficient silicon-rich dielectric and not a stoichiometric SiO_2_ subjected to the processing details [1]. The quality of the formed SiO_2_ IL significantly affects the timing of transient charging instability, which influences the preexisting defect concentration [1]. The enhanced oxygen vacancy precursor is closely related to the high-*k* metal gate process through the atomic layer deposition conditions. The postdeposition anneals during metal gate deposition process can also contribute to the boosted oxygen vacancy precursor. These reports indicate the need to investigate the properties of SiO_2_ IL further because of their effects on the resulting HfO_2_ layer and consequently on the transistor reliability.

Recent studies on dynamic NBTI stresses suggest that the degradation mechanism is related to oxygen vacancy defect (*E*′ center) [10]. The oxygen vacancy defect is the cause of hole traps in the gate oxide which are present after NBTI stress in the form of paramagnetic *E*′ or *k*_*N*_ center [11]. The dynamic NBTI measurement approach highlights that the H-related defect explained using R-D should be reviewed with regard to the inconsistency of the generation or recovery of interface trap with the model [12]. The dynamic NBTI measurement approach finds that the hole trapping effects can significantly affect the transistor reliability [10, 11]. This is attributed to the hole-trap transformation that is largely influenced by the structural relaxation which is increased at high temperatures; hence, the trapped hole site becomes further resistant to recovery [11]. In addition, it is found that greater structural relaxation of oxygen vacancy precursor site following hole captures occurs in high-*k* based devices compared to conventional SiO_2_ devices due to stronger charge-lattice interaction of more ionic properties of the HfO_2_ [11].

In contrast, the hole trapping effects found during less recovery stress-measure-stress measurement approach was made to known to be extrinsic should be excluded in the NBTI characterization [13]. This is due to the fact that the hole trapping effects give different time exponent which is inconsistent with R-D model. Following this, the separation technique is developed which gives an ideal interface trap generation behavior without hole trapping effect accounted; hence, time exponent, *n* ~ 0.16, is obtained which is consistent with R-D model. Similarly, the separation technique was also developed using ultrafast measurement for dynamic NBTI [4]. From this ultrafast measurement approach, the dynamic NBTI is shown to be determined by the cyclic hole trapping/detrapping and the relatively permanent featuring interface-state generation. The time exponent obtained was varied based on the evolution of threshold voltage shift with regard to the level of recovery voltages applied [4].

As the creation of interface traps and hole trapping in preexisting defects in the gate dielectrics leads to a reduced reliability performance, another key issue that requires attention is the dipole layer. The dipole layer is formed within the interface layer between HfO_2_ and SiO_2_ IL. The exact mechanism and origin of dipole layers are still being debated, specifically whether or not it influences the flat band and Δ*V*th [14]. The creation of a dipole layer is understood to be due to areal density difference of oxygen atoms at high-*k*/SiO_2_ interface [15]. Therefore, in this study, we incorporate the dipole interface model to investigate the relationship between Δ*V*th phenomena and NBTI stressing [16]. Our study focuses on the dipole surface density change in scaled SiO_2_ IL. The purpose of exploiting dipole interface model in this NBTI study is to investigate the correlation between the NBTI degradation and effect of dipole layer formation for high-*k* devices. To date the investigation on correlation between NBTI degradation and dipole layer formation in high-*k* devices based on NBTI simulation or experimental work has not been established.

This simulation study on NBTI is based on the two-stage NBTI model, which focuses on the creation of positively charged *E*′ center (hole trap density, *S*_2_) and dangling bond Si (interface trap/P_b_ center, *S*_4_), which are found to exist in the dynamic NBTI measurement approach briefly discussed previously. The creation of these two defects is based on variation of geometric structure and is related to high-*k* integration processes subsequent to the NBTI stressing. The characteristics will be studied based on the time evolution of the hole trap and interface trap densities, as well as the resulting Δ*V*th.

## 2. Simulation Methodology

### 2.1. Advanced-Process 32 nm High-*k* PMOS Testbed Device

**Figure 1:**
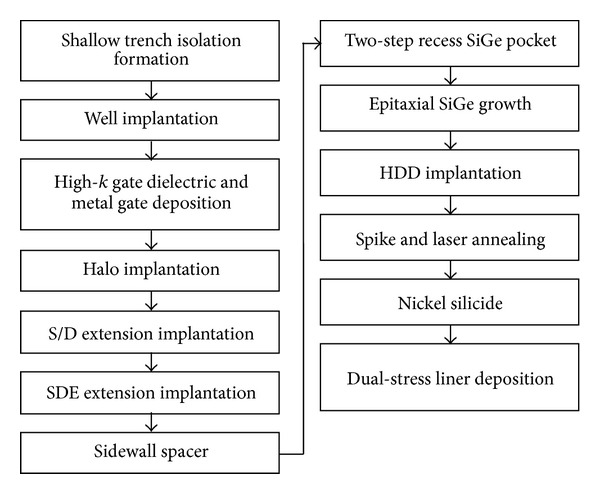
Process flow for simulated test bed device using high-*k* metal gate process of the 32 nm technology node.

The Sentaurus Synopsys TCAD simulator is used in this work. The test bed p-MOSFET devices with high-*k*/SiO_2_ gate stacks simulated in this study are based on the foundry-standard 32 nm CMOS process. The fabrication process incorporates shallow trench isolation, deposition of high-*k* dielectrics with metal gate, stress engineering using epi-SiGe pockets, silicidation, and dual-stress liner [17, 21]. The fabrication process flow used in this work is presented in Figure 1. This gate-first process scheme was adopted to overcome process-related problems (such as ultrashallow junction formation), to suppress leakage current, and to improve the drive current. The laser annealing process was incorporated to help suppress transient-enhanced dopant diffusion.

**Figure 2:**
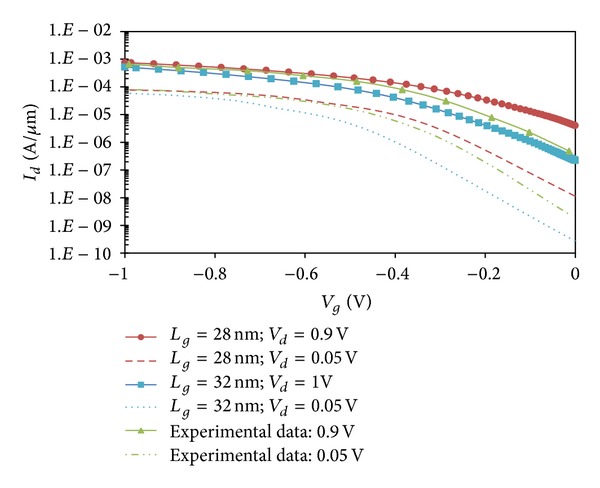
*I*_*d*_-*V*_*g*_ characteristics of simulated devices and comparison with experimental data in [17].

To validate the test bed device, the electrical characteristics of the simulated device are compared with the measured electrical characteristics of a closely similar 24 nm gate length transistor fabricated in [17], as shown in Figure 2. The key electrical parameters of the simulated device in this study are summarized and compared in Table 1. The closely similar electrical parameters between the simulated device and those of the real, physical device provides a certain degree of confidence that the impact of NBTI degradation studied in this work is realistically assessed. All subsequent simulations in this study are henceforth based on the 32 nm gate length p-MOSFET test bed device.

### 2.2. Details of Device Structure and Characterization Simulation Setup

**Figure 3:**
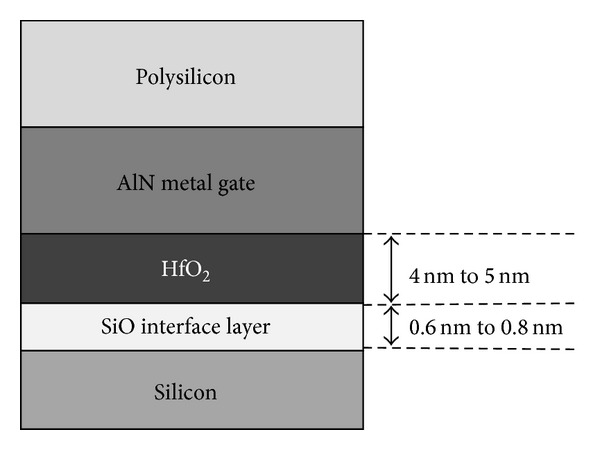
Gate stack cross-sectional profile of the testbed PMOSFET with variation in sublayers.

To examine the effects of gate geometrics on NBTI, we follow an earlier study [21] by varying the physical layer thickness of the HfO_2_ dielectric layer and the SiO_2_ IL. The thickness of each deposited stack layer is within the range of nominal thickness shown in Figure 3 [21, 22]. This study aims to account for the contribution of the hole trap to the NBTI degradation effect, whereas the earlier work explains only the interface trap generation in characterizing NBTI effects.

**Figure 4:**
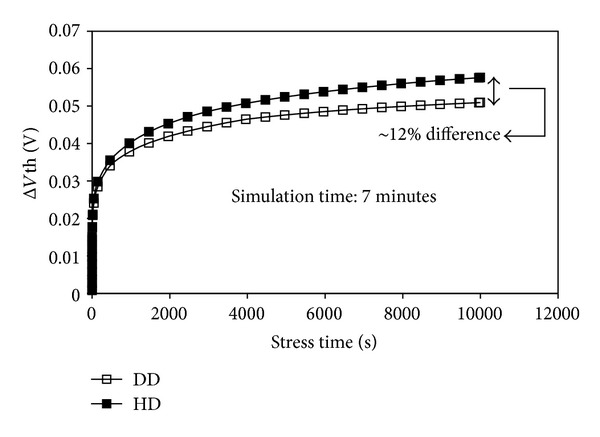
Comparison of NBTI-induced threshold voltage shift computed by hydrodynamic (HD) transport model and drift diffusion (DD) model. The DD model underestimates the degradation particularly at extended stress time.

Based on the hydrodynamic transport model, the simulator self-consistently solves the holes and electrons current continuity equations coupled to Poisson's equations. The application of the hydrodynamic transport model can better capture the effects of carrier heating in the largely-varying electric fields inside a submicron device subjected to the extreme conditions of NBTI stresses, compared to the relatively simpler drift-diffusion simulation model. This results in more physically accurate simulation results, as shown in Figure 4 which compares the degradation computed by the hydrodynamic transport model and drift diffusion. It can be seen from the figure that the drift-diffusion model underestimates the NBTI-induced Δ*V*th by as much as 12%, and the under estimation seems to be growing with extended stress time.

To model the NBTI effects on the devices, the interface regions are defined in the device structure because the trap and charge densities are defined on the interfaces by using a Sentaurus mesh [16]. The NBTI physical model is defined in the interface region between the silicon and oxide layers. The solutions of the device equations along with the two-stage NBTI model are used to extract the threshold voltage degradation, hole trap, and interface trap densities [16]. The stress temperatures range from 300 K to 400 K, which conform to the experimental settings of other NBTI studies [4, 21].

The threshold voltage degradation in this simulation is determined using the widely adopted on-the-fly (OTF) method [23–26]. Application of prestress voltage is needed in this model to equilibrate the occupancy of different states and thus ensure that all states are not empty at the early phase of stress period [18]. Time-zero delay in the OTF method introduces an artifact for the measured threshold voltage shift; thus, BTI power law is not observed during short stress [27]. Therefore, in this simulation, the stress time is increased up to 1,000 s to minimize the issue.

The two-stage NBTI model implemented in this work follows closely the default parameters similar to other works [16, 18, 19].  Table 2 shows the default parameters used in this work.

### 2.3. Model Validations

**Figure 5:**
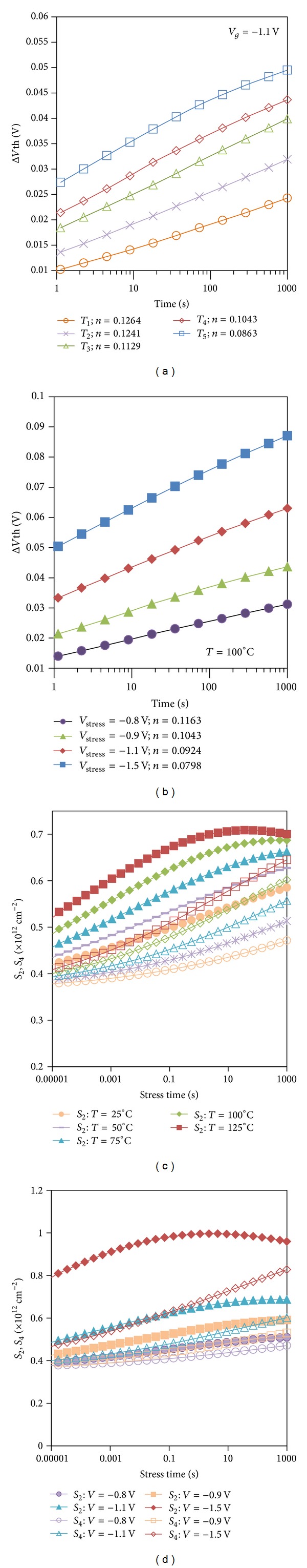
Threshold voltage shift as a function of stress time at (a) different stress temperatures and (b) different stress gate voltages. Hole trap density, *S*_2_, and interface trap density, *S*_4_, as a function of stress time at (c) different stress temperatures and (d) different stress gate voltages.

The accuracy of the two-stage NBTI model used in this study is validated by observing the power-law time dependence of the resulting Δ*V*th. The observed power-law time dependence shown in Figures 5(a) and 5(b) is subsequent to different stress temperatures and stress voltage, respectively. A relatively small exponent of *n* ~ 0.1 is obtained and attributed to the hole trapping effect. This occurrence agrees with ultrafast switching measurements in [4], which suggests that the dynamic NBTI is due to both the hole trapping/detrapping event and interface state generation.

To further examine the kinetics behind NBTI degradation effects, the hole trap density, *S*_2_, and interface trap density, *S*_4_, are plotted in Figures 5(c) and 5(d), respectively. The kinetics of the hole trap density, *S*_2_, and interface trap density, *S*_4_, observed for all the devices show almost similar behavior. The density of the hole trap, *S*_2_, is higher than that of the interface trap, *S*_4_, during the early stressing period. However, as stress time increases, the hole trap density, *S*_2_, is reduced while the interface trap density, *S*_4_, continues to increase. This mechanism, which does not comply with the hydrogen-transport model, is also reported in other studies on dynamic NBTI [4, 5], thereby lending further credibility to the two-stage NBTI model used in this study. The graphs indicate that the hole traps are gradually transformed into a more permanent form, depassivating the interface trap precursor as triggered by hole captured at an *E*′ center precursor [5]. This phenomenon has also been reported by other NBTI studies using the ultrafast measurement method [4, 5]. The time evolution of distribution of interface trap density, *S*_4_, and hole trap density, *S*_2_, shows that the degradation kinetic is more noticeable in oxide bulk than in the interface region. The degradation kinetic observed is similar under a wide range of stress voltages and temperatures.

## 3. Simulation Results

### 3.1. Gate Stack Variation

#### 3.1.1. Effects of Gate Stack Sublayer Physical Thickness on NBTI

**Figure 6:**
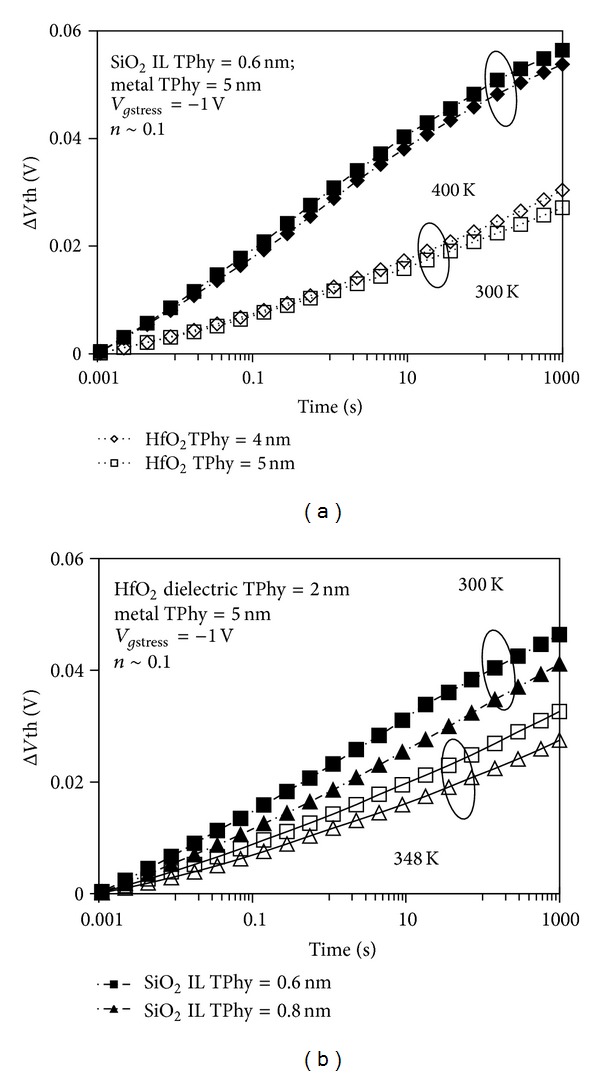
Power-law time dependence of simulated Δ*V*th for variation of physical thickness (TPhy) for (a) HfO_2_ layer and (b) SiO_2_ IL.

This section presents the simulation results of the NBTI degradation subsequent to varying the physical thicknesses of the bulk HfO_2_ and the SiO_2_ IL layer. In Figure 6(a), we compare the Δ*V*th by varying the physical thickness of the HfO_2_ layer. A thicker HfO_2_ dielectric layer at higher stress temperature exhibits larger Δ*V*th. This observation is in accordance with the measurement reported in [6], where a more pronounced Δ*V*th is observed because of more trapping centers in the thicker bulk of the HfO_2_ dielectric layer. As shown in Figure 6(b), thicker SiO_2_ IL results in less Δ*V*th, which is also in agreement with [6]. The less Δ*V*th observed in thicker SiO_2_ IL can be attributed to smaller fast transient charging effect, which is similarly reported in other studies [6, 28]. According to [13], the thinner SiO_2_ IL experiences higher gate oxide field, thereby accelerating the NBTI degradation effect because of the subsequent increase of the diffusion rate of hydrogen species. In this study, the contribution of hole trap density to the degradation is computed. Thus, the higher gate oxide field accelerates the hole trapping effect, which increases the degradation rate. The contribution of the hole trap density, *S*_2_, and interface trap density, *S*_4_, to the degradation of scaled SiO_2_ thickness will be described in subsequent sections of this paper.

#### 3.1.2. Effects of Scaled SiO_2_ Interface Layer Thickness during Stress and Recovery Cycle

**Figure 7:**
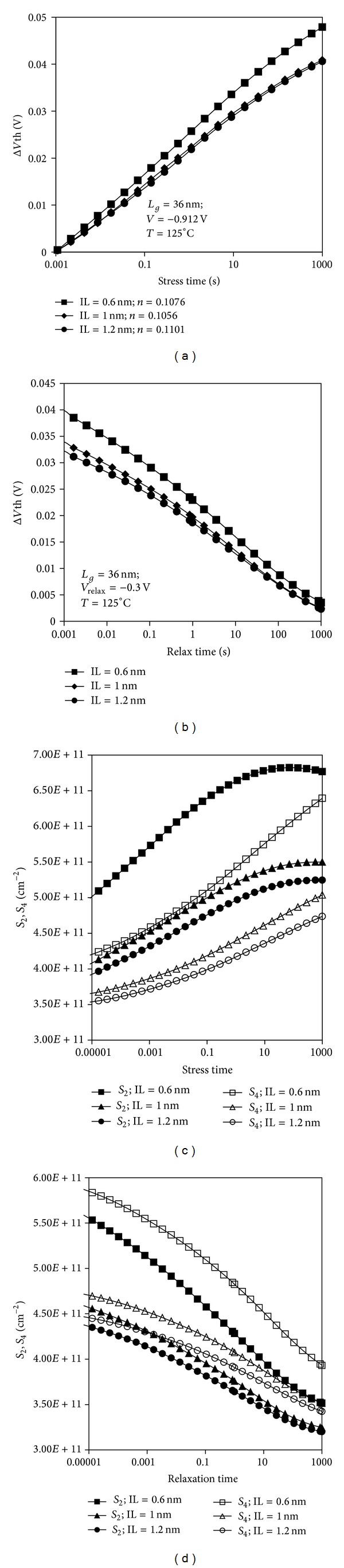
Threshold voltage shift for different SiO_2_ IL thickness during the (a) stress phase and (b) relaxation phase as a function of stress and relaxation time, respectively. Time evolution of hole trap density, *S*_2_, and interface trap density, *S*_4_, for different SiO_2_ IL thickness during the (c) stress phase and the (d) relaxation phase as a function of stress and relaxation time, respectively.

After validating the accuracy of the two-stage model in modeling the kinetics of electrically active defects in high-*k* PMOSFET device subsequent to wide range of bias and temperature stress condition, we further conducted simulation to study detailed behavior of NBTI degradation. This study was conducted by varying the stress and recovery condition of PMOSFETs with different SiO_2_ IL thicknesses. The time evolution of threshold voltage shift was studied during the stress and relaxation phase as shown in Figures 7(a) and 7(b), respectively. Higher degradation is observed in thinner SiO_2_ IL, as discussed previously. The relaxation phase dynamics follow the stress characteristics such that thinner SiO_2_ exhibits slower relaxation effects.

The dynamic behavior of stress and relax mechanisms are further explained by the time evolution of the hole trap density, *S*_2_, and interface trap density, *S*_4_, as shown in Figures 7(c) and 7(d), respectively. The hole trap density, *S*_2_, and interface trap density, *S*_4_, are higher in the device with thinner SiO_2_ IL. The higher hole trap density, *S*_2_, in thinner IL device could be explained by the diffusion of oxygen from IL during the deposition process of HfO_2_ on IL [8]. All of the fabrication process parameters used in the simulation were fixed except for the IL thickness, which suggests that more oxygen is diffused in thinner IL; thus, higher density of *E*′ defects is expected. This finding concurs with the ESR measurement study on electrically active IL defects, in which *E*′ center defects function as hole traps [7]. The defects can act as hole traps because of their locations, which are close to the Si/dielectric interface and allows the defects to play a role in the charge capture. These *E*′ defects increase during stress bias upon the capturing of holes from the channel, as explained by the switching hole trap mechanism. The depassivation of the interface trap precursor which is triggered by the hole capturing process at the *E*′ center increases as stress time increases. This phenomenon is observed in most studies using the ultrafast measurement method [4, 5].

The stress time at which the hole trap density, *S*_2_, is reduced for different thicknesses of SiO_2_ IL constantly occurs after 10 s stress duration. This occurrence implies that the hole traps take a more permanent form and do not depend on the SiO_2_ IL thickness for a particular applied stress voltage and temperature. As indicated in Figure 7(c), within the 1,000 s stress time, the hole trap density, *S*_2_, is always higher than the interface trap density, *S*_4_. One can deduce that, during the period of stress, the defects are more significant in the oxide bulk than at the interface between the oxide and substrate. However, approaching the end of stress time, the positively charged *E*′ centers are reduced significantly, whereas the interface traps continue to increase.

During the relaxation phase, interface trap density, *S*_4_, is higher than the hole trap density, *S*_2_, and both are reduced as the relaxation time increases. This behavior is in agreement with the experimental work in [29] based on DCIV measurement, where hole trap density, *S*_2_, decreases during relaxation through a repassivation process of interface traps. Higher interface trap density, *S*_4_, during the relaxation process occurs when hydrogen leaves behind unpassivated dangling bond locked in the positively charged *E*′ center defect, which effectively delays the recovery of the *E*′ center defect [30, 31]. These defects are completely discharged when the stress bias is removed [4]. However, the discharge process is highly dependent on time at which relaxation phase dynamics is slower for thicker SiO_2_ IL. This dependence may be ascribed to slow charging kinetics of the defects in thicker SiO_2_ IL, as highlighted previously.

#### 3.1.3. Effects of Gate Stack Sublayer Physical Thickness on Recoverable Component

**Figure 8:**
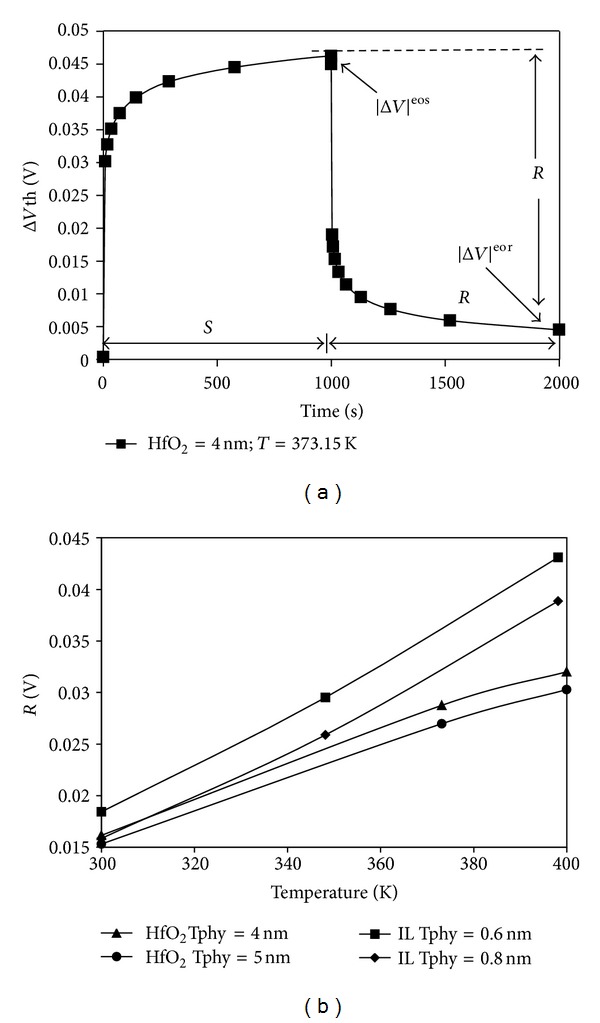
(a) Evolution of Δ*V*th and definition of recovery component, *R*, in a typical dynamic NBTI cycle and (b) *R* as a function of stress temperature.

Figure 8(a) shows the evolution of dynamic NBTI as a result of a simulated stress and relax cycle. The hole trap transformation can be further explored by analyzing the |Δ*Vt*| recovered per cycle, *R*, as determined in the figure, by following the work in [11]. As shown in Figure 8(b), *R* is a function of stress temperature for devices with variation in HfO_2_ dielectric layer and SiO_2_ IL thicknesses. The relationship obtained between *R* and stress temperature, as illustrated in Figure 8(b), suggests that the hole transformation is thermally activated. This activation occurs because, during higher stress temperature, the hole trapping effect is increased, which is in agreement with [11]. A closer observation indicates that more recoverable component can be observed in thinner SiO_2_ and HfO_2_.

### 3.2. High-*k* Process Integration Issue

In this section, we discuss the influence of related reliability issues in the gate insulator processing in high-*k* metal gate devices, namely, the oxygen vacancy precursor and dipole charge. We discuss the effects of different densities of oxygen vacancy precursors and dipoles on the NBTI degradation. This study is conducted for PMOSFETs with varied thicknesses of dielectric layers.

#### 3.2.1. Correlation of Gate Stack Sublayer Physical Thickness and Oxygen Vacancy Precursor Concentration on NBTI

**Figure 9:**
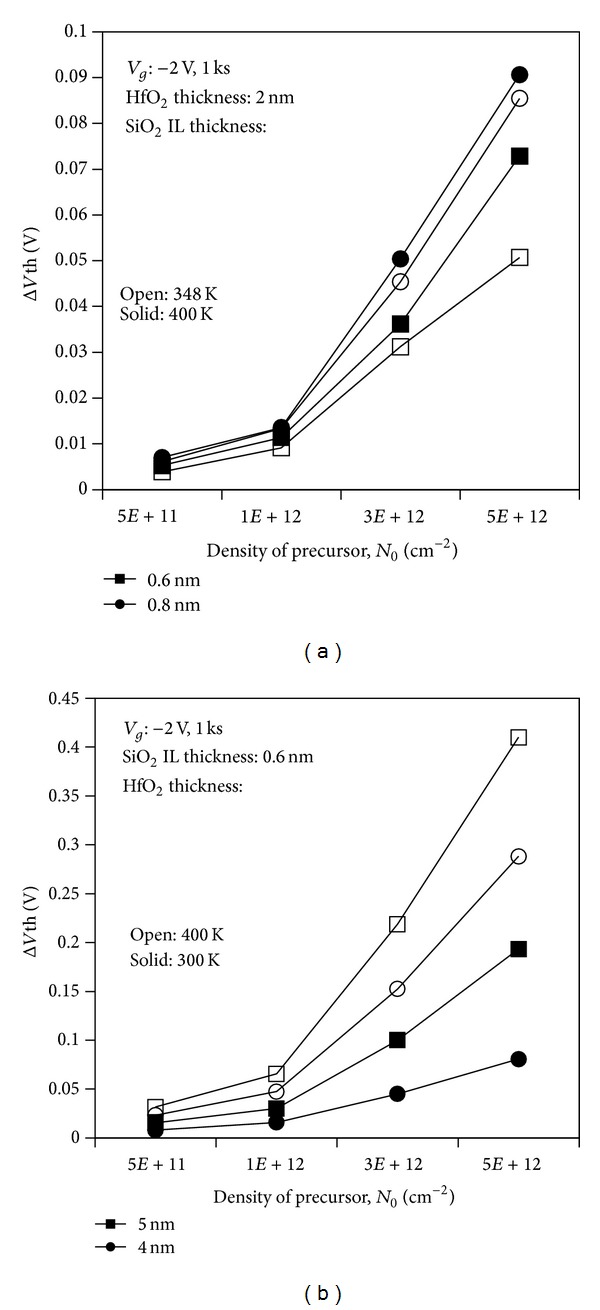
Evolution of threshold voltage shift based on different densities of precursor. (a) Variation in thicknesses of SiO_2_ with thickness of 2 nm HfO_2_. (b) Variation in thicknesses of HfO_2_ with thickness of 0.6 nm SiO_2_ interface layer.

Figures 9(a) and 9(b) illustrate *V*th shift kinetics according to the density of precursors. This study has been conducted on PMOSFET devices with varied thicknesses of the SiO_2_ interface and HfO_2_ layers. All devices in both figures show that the degradation occurs more in the case with high density of precursor. Higher degradation is observed during higher stress temperature for all devices. As stated, the oxygen vacancy precursor in the oxide can significantly generate the hole trap in the oxide upon the application of stress bias. Higher density of oxygen vacancy precursor leads to more hole traps in the oxide, which enhances the degradation level.

However, contrary to the enhanced degradation upon scaled SiO_2_ interface layer thickness in the previous discussion, Figure 9(a) shows a different result. The thicker SiO_2_ interface layer leads to more degradation under higher applied stress voltage. This degradation could be attributed to the higher amount of hydrogen available in thicker SiO_2_ interface layer [32]. In the two-stage NBTI model, the positively charged *E*′ center can attract the H from P_b_H, thereby creating the dangling bond at the interface [20, 30]. Higher stress voltage produces more positively charged *E*′ centers and can therefore attract more H, which subsequently creates more dangling bonds at the interface.

In Figure 9(b), the increased trapping occurs at preexisting defects in thicker high-*k* which is similar to what has been observed in the previous section. For the fixed SiO_2_ interface layer, a similar amount of H takes part in the degradation mechanism for each device, which further proves that the occurrence of degradation is increased in the trapping process in preexisting defect. Thus, more positively charged *E*′ centers are created for thicker HfO_2_ dielectric upon the application of higher stress voltage. As indicated in Figure 9(b), severe NBTI degradation for device with 5 nm HfO_2_ thicknesses occurs at higher stress voltage, such that this device experiences more than 10% *V*th shift for the oxygen precursor of 5 × 10^12^ cm^−2^ at room temperature and higher. Devices with thinner HfO_2_ in Figure 9(a) exhibit less than 10% *V*th shift for oxygen precursor of 5 × 10^12^ cm^−2^ at higher stress voltage application. We can thus conclude that the optimization of HfO_2_ thickness is crucial in order to minimize the NBTI degradation effect. The density of the oxygen precursor should be approximately 1 × 10^12^ cm^−2^ or less for devices with HfO_2_ thicknesses of 4 nm and 5 nm to ensure a *V*th shift of not more than 10% during the specified stress duration. The severe degradation observed in high-*k* metal gate devices with thicker high-*k* dielectric implies that the defect mechanisms are more significant in the oxide bulk compared with the interface between oxide and substrate.

**Figure 10:**
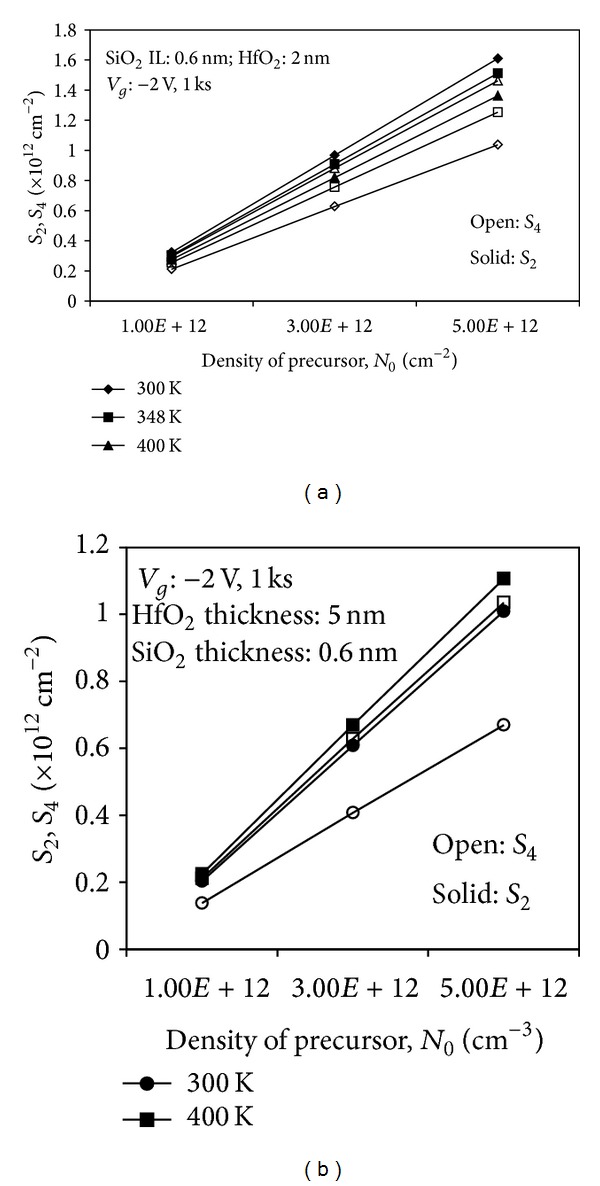
Hole trap density, *S*_2_, and interface trap density, *S*_4_, as a function of density of precursor under a different stress temperature for (a) thin HfO_2_ and (b) thick HfO_2_.

In Figures 10(a) and 10(b), the hole and interface trap densities (*S*_2_ and *S*_4_) are shown as a function of density of the precursor for devices with thinner and thicker HfO_2_, respectively, under a different stress temperature. The first impression on the number of hole trap density, *S*_2_, and interface trap density, *S*_4_, in both graphs is that the defects increase as the density of oxygen vacancy precursor increases. However, a closer observation indicates that the dependence of the generated hole trap density, *S*_2_, and interface trap density, *S*_4_, on temperature is contradicted with a different thickness of HfO_2_. For devices with thinner HfO_2_, the generated positively charged *E*′ centers are insensitive to stress temperature. The hole trap density, *S*_2_, is larger at room temperature and decreases as the stress temperature is increased after 1,000 s stress. As the thinner HfO_2_ has less trapping in the bulk high-*k* layer [6], the reduction in the generated hole trap density at higher stress temperature is observed. This reduction is due to the transformation of the hole trap density, *S*_2_, into the interface trap density, *S*_4_, in accordance with constant voltage stress measurement in [10]. As shown in this study, the interface trap density, *S*_4_, is considered as a permanent oxide trap. This transformation process is said to happen during an adequately long duration of* dc* NBTI stress [10]. The reduction of generated trapped holes at higher stress temperature implies that a more permanent oxide trap is created after the 1,000 s stress in accordance with the experiment. The thermally activated transformation of the hole trap density, *S*_2_, into the permanent oxide trap is observed as more interface trap density, *S*_4_, is created at higher stress temperature.

By contrast, for devices with thicker HfO_2_, the higher degradation is observed, as discussed in the previous section. This degradation could be attributed to the higher generated hole trap upon application of bias resulting from fast transient charging effect [6]. Thus, the transformation of the permanent hole trap has an increased probability of occurrence. The generated interface trap density, *S*_2_, at room temperature is considerably smaller than the generated interface trap density, *S*_4_, at higher temperature.

**Figure 11:**
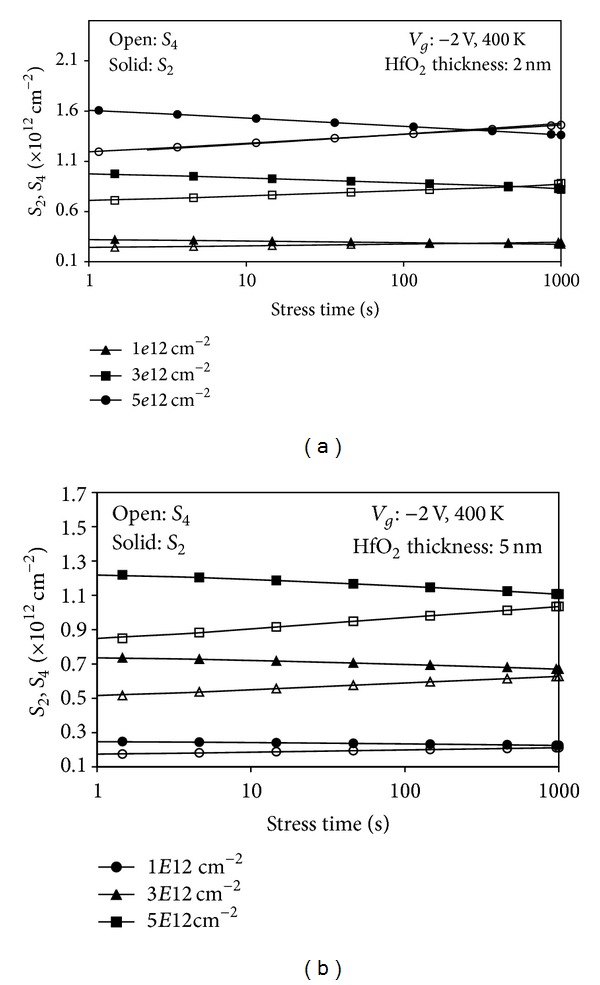
Time evolution for hole trap density, *S*_2_, and interface trap density, *S*_4_, for a different number of precursors. (a) Thin HfO_2_ and (b) thick HfO_2_.

Figures 11(a) and 11(b) show the time evolution of hole and interface trap densities (*S*_2_ and *S*_4_) based on a different number of precursors for thin HfO_2_ and thick HfO_2_ layers, respectively. A thinner HfO_2_ layer shows that, at high density of the precursor, the degradation is more prominent for interface traps than with those in bulk oxides as stress time increases. This degradation is due to more interface trap density, *S*_4_, than the hole trap density, *S*_2_, created. By contrast, for a thicker HfO_2_ layer, the hole trap density, *S*_2_, is greater than the interface trap density, *S*_4_, within the specified stress time. Therefore, this greater density further confirms that a thicker HfO_2_ layer contributed to increased degradation, and the occurrence of the degradation is more prominent in the oxide bulk. A closer observation indicates that, during earlier stress time, the degradation kinetics occur more often in the oxide bulk, as the hole trap density is more than the interface trap density. At shorter stress times, the number of precursors, which influences the degradation, contributes more to the degradation of the thinner HfO_2_ layer. This phenomenon is attributed to the higher number of precursors, which increases the number of holes that can be trapped upon the application of stress bias. This increase in turn enhances the creation of interface trap density, *S*_4_, as explained by the two-stage model mechanism [20, 30]. By contrast, with regard to the thicker HfO_2_ layer, the higher number of precursors led to a longer stress time needed for the interface trap density, *S*_4_, to influence the degradation mechanism further. This appearance can be explained with regard to the influence of different thicknesses of HfO_2_ layer deposited on a similar thickness of SiO_2_ interface layer as discussed in [3]. More oxygen vacancies are generated at the SiO_2_/Si interface and in the bulk IL for thicker HfO_2_, as observed in the experimental work. We conclude that, for the thicker HfO_2_ layer, more oxygen vacancies are available; thus, the higher defined number of precursors significantly influences the degradation mechanism. As a result, greater hole trap density, *S*_2_, than interface trap density, *S*_4_, occurs throughout the specified stress time. This occurrence results in more degradation in the bulk oxide than in the interface region.

#### 3.2.2. Correlation of Scaled SiO_2_ Interface Layer Physical Thickness and Dipole Layer on NBTI

**Figure 12:**
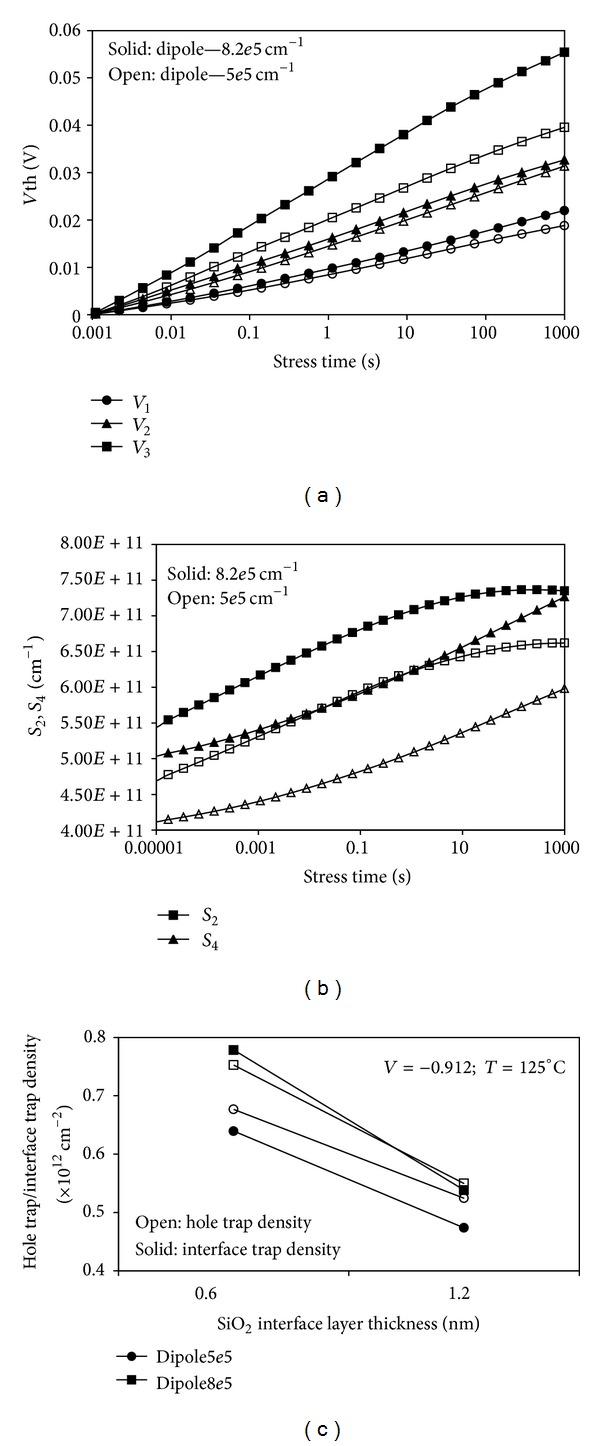
(a) Threshold voltage shift (*V*_1_ < *V*_2_ < *V*_3_) and (b) time evolution of hole and interface trap densities because of the effect of different dipole surface densities. (c) Hole and interface trap densities in relation to different SiO_2_ interface layer thickness for a different dipole layer.

This section highlights the relationship between dipole surface density and the NBTI degradation effect. The two-stage NBTI model is further used to explain the NBTI degradation effect with the influence of dipole surface density. For a similar stress bias condition, the higher dipole surface density influences the threshold voltage shift more than the lower dipole surface density, as depicted in Figure 12(a). Dipole charges formed between the SiO_2_ IL and HfO_2_ layers produce electrostatic potential that can influence the dynamic behavior of the hole and interface trap densities (*S*_2_ and *S*_4_), as indicated in Figure 12(b). The occurrence of the degradation mechanism is not influenced by the change in dipole surface density. The degradation is more significant in the oxide bulk than in the interface region. To investigate the effects of different dipole surface densities on varied SiO_2_ IL thicknesses, the hole and interface trap densities are plotted after the 1,000 s stress, as shown in Figure 12(c). Higher density of the hole trap, *S*_2_, is observed for higher dipole surface density in thinner SiO_2_ IL. Similarly, the density of the interface trap, *S*_4_, is also higher when the dipole surface density is higher.

For devices with thin and thick SiO_2_ interface layers, more pronounced degradation is observed in the oxide bulk as the hole trap density, *S*_2_, is higher when the dipole layer is smaller. By contrast, Figure 12(c) shows that for a larger dipole layer, the location of the degradation mechanism is different for devices with thin and thick SiO_2_ interface layers. For thinner SiO_2_ interface layers, the degradation mechanism occurred more in the interface, whereas for thicker SiO_2_ interface layers, the degradation mechanism occurred slightly more in the oxide bulk.

One of the possible explanations for the relationship between the threshold voltage shift and the dipole layer effect can be attributed to the effect of the dipole on channel mobility. The channel mobility decreases with thinning SiO_2_ IL because of remote scattering that occurs as a result of high-*k* phonon scattering and coulomb scattering caused by oxide charges in the high-*k* layer [33]. In principle, in the bilayer structure consisting of two dielectric materials with different conductivities, the charge trapping at the interface occurs as predicted in electromagnetic characteristics. Considering the existence of defects at the interface, we believe that these defects play an important role in the charge trapping. Therefore, the effects of dipole layer formation between the high-*k* and SiO_2_ layers should be considered in assessing the NBTI degradation effect. However, the dipole layer formation strongly depends on the applied voltage and other charges between the gate and substrate, including ionized donors and acceptors. Therefore, specific measurement should be developed to characterize the influence of the dipole layer on the NBTI degradation.

## 4. Conclusion

We have observed that the applied stress voltage and physical thickness of the dielectrics in the high-*k* metal gate stack in PMOSFETs exerts a certain influence on the degree of NBTI degradation. The degradation decreases for thicker SiO_2_ IL under small stress voltage and increases under high stress voltage application. However, the degradation decreases for thinner HfO_2_ and is not influenced by the stress voltage levels. The occurrence of the defect mechanism in the oxide bulk or interface region is also influenced by the stress conditions, physical thickness of the dielectrics, and number of precursor and dipole layer charges. The transformation of hole trapping into permanent trapped holes is thermally activated, as proven by the recoverable component, *R* behavior. Optimizing the gate stack dielectric sublayer physical thickness is essential to achieve a more reliable NBTI and high-*k* gate stack aging model.

## Figures and Tables

**Table 1:** Performance summary of simulated devices.

	This study		[17]
*L*_*g*_ (nm)	28	32	24
*V*_dd_ (V)	0.9	1	0.9
*I*_*on*⁡_ (A/um)	7.53 × 10^−4^	5.16 × 10^−4^	5.25 × 10^−4^
*I*_*off*⁡_ (A/um)	3.97 × 10^−6^	2.22 × 10^−7^	3.70 × 10^−7^

**Table 2:** Default spread of energy wells and barriers used in simulation [16, 18–20].

Parameter	Definition	Default values (eV)
*E*_1_	Trap level of the precursor	−1.14 to −0.31
*E*_2_	Trap level of the *E*′ center	0.01 to 0.3
*E*_4_	Trap level of the P_b_ center	0.01 to 0.5
*E*_*A*_	Barrier energy of a transition from state 3 to state 1	0.01 to 1.15
*E*_*B*_	Barrier energy of a transition from state 1 to state 2	0.01 to 1.15
*E*_*D*,std-dev_	Barrier energy of a transition from state 2 to state 4	0.44 eV

Parameter	Definition	Default values

*σ*_*n*_	Electron cross-section	1.08 × 10^−15^ cm^2^
*σ*_*p*_	Hole capture cross-section	1.24 × 10^−15^ cm^2^
*γ*	Prefactor for field-dependent barrier energy	0.74 nm
*v*_1_	Attempt frequencies	10^13^ s^−1^
